# Suspected “T-Cell-Mediated” Hypereosinophilic Syndrome Presenting with Cerebral Watershed Infarcts

**DOI:** 10.1155/2011/834308

**Published:** 2011-11-29

**Authors:** S. Dujardin, R. Schots, S. De Raedt

**Affiliations:** ^1^Department of Neurology, Universitair Ziekenhuis Brussel, Vrije Universiteit Brussel, 1090 Brussels, Belgium; ^2^Department of Clinical Hematology, Universitair Ziekenhuis Brussel, Vrije Universiteit Brussel, 1090 Brussels, Belgium

## Abstract

We describe a case of suspected “T-cell-mediated” hypereosinophilic syndrome presenting with cerebral watershed infarcts. An extensive search for potential embolic sources was negative, supporting the hypothesis that cerebrovascular endothelial dysfunction could have caused the infarcts.

## 1. Introduction

Watershed infarcts are ischemic lesions in the junctions of the distal fields of two nonanastomosing arterial systems (border zones). The pathogenesis of watershed infarction is still a matter of debate. Traditional causes are severe hemodynamic restriction (due to systemic hypotension or major vessel stenosis/occlusion) and emboli. The more recent mixed emboli-hemodynamic hypothesis states that the underlying hemodynamic impairment favors the development of infarcts in the border zones when small emboli reach the distal field [1].

Watershed infarcts also have been associated with hypereosinophilia in case reports. Major proposed mechanisms were distant microemboli from endocardial fibrosis [2] and local cerebrovascular thrombi [2, 3].

## 2. Case Report

A 56-year-old man presented to the Emergency Department with complaints of headache, clumsiness of his right hand, and numbness in the right side of his face, which had suddenly developed a few days earlier.

The patient had a history of hypertension (treated with carvedilol, lisinopril, barnidipine, and moxonidine), chronic renal insufficiency, eczema, and three episodes of bacterial pneumonia.

Other daily medications were acetylsalicylic acid 100 mg and simvastatin 20 mg.

On examination, body temperature was 35.9°C, pulse was 94/min, and blood pressure was 162/95 mmHg.

Neurological examination revealed a zone of reduced sensitivity in the right face, not confined to a trigeminal nerve dermatome. Strength and coordination of the right hand were reduced. Tendon reflexes were weak. Plantar reflexes were normal. The patient was able to walk and stand normally, but was mentally slow and showed apraxia.

Laboratory examination revealed an increased C-reactive protein (151 mg/L), a mild anemia, and a moderate hypereosinophilia (3.5 × 10^3^/mm^3^). Cardiac troponins I were slightly elevated (3.41 *μ*g/L) with normal creatine kinase-MB levels. A coagulation screen including anticardiolipin and lupus anticoagulant revealed no abnormality.

**Figure 1:**
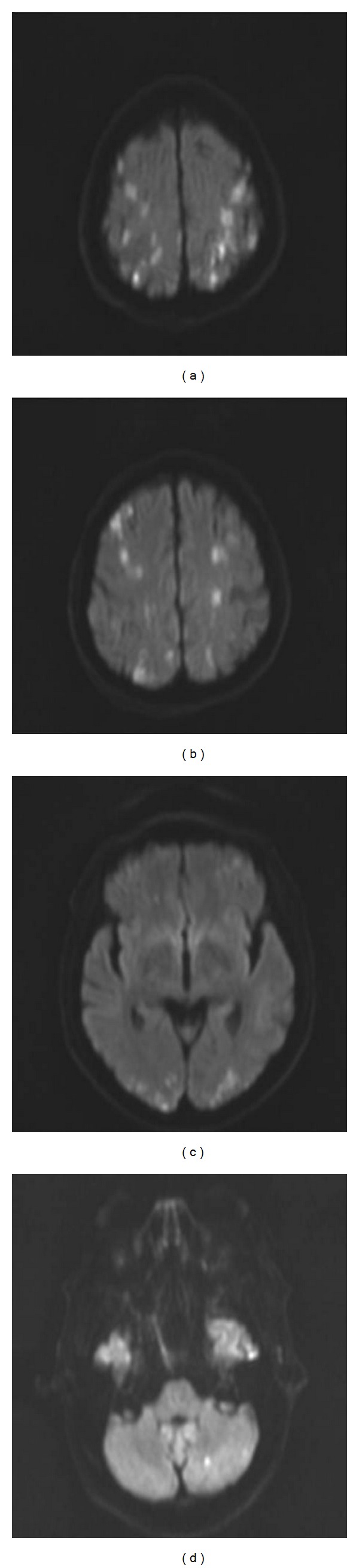
Diffusion-weighted imaging shows multiple zones of diffusion restriction in the watershed regions of both cerebral hemispheres and in the left cerebellar hemisphere.

Brain MRI showed several small areas of diffusion restriction in both cerebral hemispheres and a single lesion in the left cerebellar hemisphere, corresponding to border-zone infarctions (Figure 1).

An extensive search for potential cardioembolic sources with ECG, 48-hour cardiac rhythm monitoring, transthoracic and transoesophageal echocardiography, cardiac MRI, and CT angiography of the thoracic aorta was negative. There were no microembolic signals detected on transcranial Doppler monitoring. Cerebrospinal fluid analysis was normal.

The patient had no history of allergic disease. Repetitive coproculture and antibodies against strongyloides, filariae, and toxocara were negative. CT thorax, CT abdomen, whole-body PET with [18F]*-*fluoroglucose, and lymphocyte immunophenotyping were not suggestive of a lymphoma. There were no history of asthma and no granulomatous skin lesions, and ANCAs were negative—findings not suggestive of a Churg-Strauss syndrome. Angiotensin-converting enzyme, ANA, and rheumatoid factor were also negative. The patient had high IgE serum levels (8770 kIU/L).

A bone marrow biopsy showed no signs of myeloproliferative disorders. The bone marrow karyotype was normal (46, XY), and FISH analysis did not show a PDGFRB rearrangement. The FIP1L1-PDGFRA fusion gene was not detected. However, a T-cell receptor rearrangement was detected by PCR on peripheral blood and bone marrow.

The patient was treated with 300 mg acetylsalicylic acid and 32 mg methylprednisolone per day. Eosinophils rapidly declined, and corticoids were tapered and withdrawn within a few weeks. Six months after initial presentation, the patient was asymptomatic, eosinophil levels were lower than 0.5 × 10^3^/mm^3^, and brain MRI showed no new lesions.

## 3. Discussion

Hypereosinophilic syndromes (HESs) are a group of disorders characterized by persistent hypereosinophilia (>1500 per *μ*L) not due to parasitic infection, allergic disease, nor other underlying disease, and with eosinophil-mediated dysfunction of at least one target organ or tissue [4].

There are different subtypes of HES [4]. In our case, we detected an abnormal population of T cells on the basis of T-cell receptor rearrangement by PCR analysis, while lymphocyte immunophenotyping remained normal. Therefore, the most likely origin of the hypereosinophilia was a suspected “T-cell-mediated” hypereosinophilic syndrome.

To our knowledge, this is the first case of watershed infarcts associated with a suspected “T-cell-mediated” hypereosinophilic syndrome.

A few other cases of watershed infarcts associated with moderate to severe hypereosinophilia have been reported. Proposed pathophysiological mechanisms include microemboli from endomyocardial fibrosis [2] or local thromboses due to eosinophil-induced endothelial dysfunction of cerebral vessels [2, 3].

Our patient had an extensive search for potential cardioembolic sources which was negative. In particular, there was no endocardial fibrosis on cardiac MRI, and emboli detection using transcranial Doppler ultrasonography was negative.

We suggest that in our case cerebrovascular endothelial dysfunction could have caused the infarcts. Similarly to the hypothesis that impaired clearance of emboli plays a role in the watershed localization of embolic ischemic strokes [1], the endothelial injury and procoagulation effect of eosinophils [5] might have a greater impact on border zones due to diminished washout of eosinophil-derived mediators. An alternative origin of the infarcts would be undetected cardiac emboli despite extensive search.
